# Structural and Quantitative MRI Signal Features in Pediatric Focal Cortical Dysplasia: Clinical Relevance

**DOI:** 10.33549/physiolres.935824

**Published:** 2026-06-01

**Authors:** Zuzana HOLUBOVÁ, David KALA, Václav ČAPEK, Nora PROFANTOVÁ, Barbora SPLÍTKOVÁ, Jan ŠANDA, Yeva PRYSIAZHNIUK, Kateřina JIRÁNKOVÁ, Martin KUDR, Pavel KRŠEK, Martin KYNČL, Jakub OTÁHAL

**Affiliations:** 1 Department of Medical Imaging, Second Faculty of Medicine, Charles University and Motol University Hospital, Prague 5, Czech Republic; 2 Department of Pathophysiology, Second Faculty of Medicine, Charles University, Prague 5, Czech Republic; 3 Department of Paediatric Neurology, Second Faculty of Medicine, Charles University and Motol University Hospital, Prague 5, Czech Republic; 4 Department of Radiology and Nuclear Medicine, Third Faculty of Medicine, Charles University and University Hospital Královské Vinohrady, Prague 10, Czech Republic; 5 Epilepsy Research Center Prague, EpiReC, Prague, Czech Republic

**Keywords:** Focal cortical dysplasia, Signal intensity, Structural MRI, Pediatric, Quantitative T1

## Abstract

The aim of this study was to quantitatively characterize MRI signal-intensity (SI) abnormalities in focal cortical dysplasia (FCD) and to compare expert visual lesion delineation with objective SI metrics across conventional structural MRI sequences and quantitative T1 mapping (qT1). Thirteen pediatric patients with histologically confirmed FCD type I or II underwent presurgical MRI including 3D T1-weighted, T2-weighted, FLAIR, and qT1 sequences. Two expert-defined lesion masks were generated: a conservative feature-focused mask (L1) and a broader contextual mask reflecting overall radiological judgement (L2). Signal-intensity values were extracted from lesions, perilesional zones, and contralateral homologous regions, and spatial correspondence with the resection cavity was evaluated using the Dice score. L2 delineations were substantially larger and showed markedly higher overlap with resected tissue than L1, suggesting that expert contextual assessment captures clinically relevant but quantitatively subtle abnormalities. In contrast, L1 showed stronger and more consistent SI abnormalities, particularly on T2-weighted and FLAIR images, indicating that expert visual judgement and quantitative SI metrics do not fully converge. Perilesional tissue closely resembled healthy brain, with only minimal SI gradients beyond the lesion border. Quantitative T1 mapping provided limited additional value over conventional T1-weighted imaging for the detection of white-matter abnormalities. These findings indicate that expert assessment and quantitative MRI capture complementary but non-identical aspects of FCD pathology, and suggest that future automated detection models should combine quantitative features with multiple layers of radiological expertise.

## Introduction

Focal cortical dysplasia (FCD) is among the most common causes of drug-resistant epilepsy in children [1–3]. Epilepsy surgery remains the only curative treatment capable of achieving long-term seizure freedom and early surgical intervention is associated with improved cognitive development, psychosocial function, and quality of life [4–6]. Postsurgical outcomes vary across histopathological subtypes and strongly depend on precise lesion localization and complete resection, with FCD type II generally yielding more favorable results than type I [4,7–11]. Accurate detection and characterization of FCD lesions are therefore crucial for pre-surgical planning and prognostic assessment [4,7–10]. However, many lesions remain MRI-occult or subtle on conventional imaging [12–14].

Conventional MRI features of FCD include cortical thickening, blurred grey-white matter junction, abnormal gyral/sulcal morphology, and subcortical white-matter signal changes, sometimes accompanied by a transmantle sign in type II lesions [3,15–18]. Signal-intensity abnormalities are commonly observed as cortical and subcortical T2w and FLAIR hyperintensity, sometimes accompanied by T1w hypointensity [15,16,19–21]. However, some lesions, particularly FCD type I, show only subtle or ambiguous changes, contributing to inter-reader variability and frequent MRI-negative cases [17,22–24].

Advanced imaging methods, including quantitative MRI (such as T1 mapping), diffusion-based models, and machine-learning approaches, can reveal subtle abnormalities not visible on conventional MRI, although their integration into routine clinical workflows remains limited [25–36]. FDG-PET offers complementary metabolic information useful especially in MRI-negative cases but does not independently define the epileptogenic lesion [23,37].

A major unmet need is a robust, objective framework for defining the true imaging extent of FCD-related abnormalities and their margins. Expert neuroradiological assessment implicitly integrates two different “read-outs”: morphological cues (e.g., blurring, cortical thickness, gyral/sulcal distortion) and a distinct intensity-based judgement, how abnormal tissue appears across sequences, which may better reflect underlying tissue composition. Yet the intensity features that actually drive expert-defined borders remain poorly standardized and have not been systematically linked to quantitative signal metrics, leaving it uncertain whether abnormalities are confined to the visually defined lesion core or extend into adjacent perilesional tissue [20,38].

Against this background, we hypothesized that expert-defined lesion margins are most closely approximated on conventional T1-weighted images, but that quantitative T1 mapping can provide a more standardized, intensity-based representation of FCD-related abnormalities and sharpen lesion-to-tissue contrast. We also hypothesized that intensity abnormalities may not be restricted to the visually defined core, but could extend into perilesional tissue. Accordingly, our goal was to map the visually defined lesion extent onto objective MRI signal metrics and to quantify potential gradients of abnormality from the lesion core to surrounding tissue.

## Methods

### Study Design

This retrospective single-center study was conducted at Motol University Hospital (Prague, Czech Republic) between 2021 and 2024. A subset of patients had previously been included in other MRI studies by our group [7,27,36] but the present analysis focuses exclusively on epileptogenic lesion signal abnormalities.

### Study cohort

Pediatric patients with drug-resistant focal epilepsy with histologically proven FCD type I or II (according to ILAE classification from 2011, resp. 2022) [2,39] were enrolled from the cohort described above. Inclusion criteria were pediatric age (0–18 years), histologically confirmed FCD I/II, MRI-visible lesion, and completion of the epilepsy-specific MRI protocol. Exclusion criteria were histopathological diagnoses other than FCD type I or II.

The extent of resection was in all cases determined by a multidisciplinary team based on integrated clinical information and multimodal diagnostic data, including seizure semiology, electrophysiological findings (scalp EEG and, when indicated, intracranial SEEG), and multimodal neuroimaging, which consistently included FDG-PET and, in selected cases, SISCOM. All patients subsequently underwent intraoperative electrocorticography, which was used to adjust the resection margins. The minimum follow-up duration was 12 months. Clinical variables included demographics, epilepsy history, seizure characteristics, and postoperative outcome.

### MRI acquisition

All MRI examinations were performed at Motol University Hospital, each patient underwent at least two scans: one pre-surgical and one post-surgical. Young or uncooperative pediatric patients (n=7) were examined under general anesthesia following institutional safety protocols.

Pre-surgical imaging was acquired on a 3 T Siemens Magnetom Vida with a 64-channel head coil using an epilepsy-optimized protocol, consistent with current HARNESS-MRI recommendations [40]. The protocol comprised: 3D T1-weighted (T1w), 3D T2-weighted (T2w) and 3D FLAIR images, and quantitative T1 mapping (qT1). Detailed acquisition parameters are provided in Supplementary Table 1, and the full protocol has been previously published [27,36,41]. First post-surgical MRI was performed on a 1.5 T Philips Achieva the day after surgery and included 3D T1w, 3D T2w, 2D T2w and FLAIR sequences (beside others) used for delineation of the resection cavity.

### MRI analysis

MRI data of structural sequences T1w, T2w and FLAIR and qT1 were processed and then used by reading radiologists as source volumes for manual segmentation of visible lesions in 3D Slicer (http://www.slicer.org) [42].

**Fig. 1:**
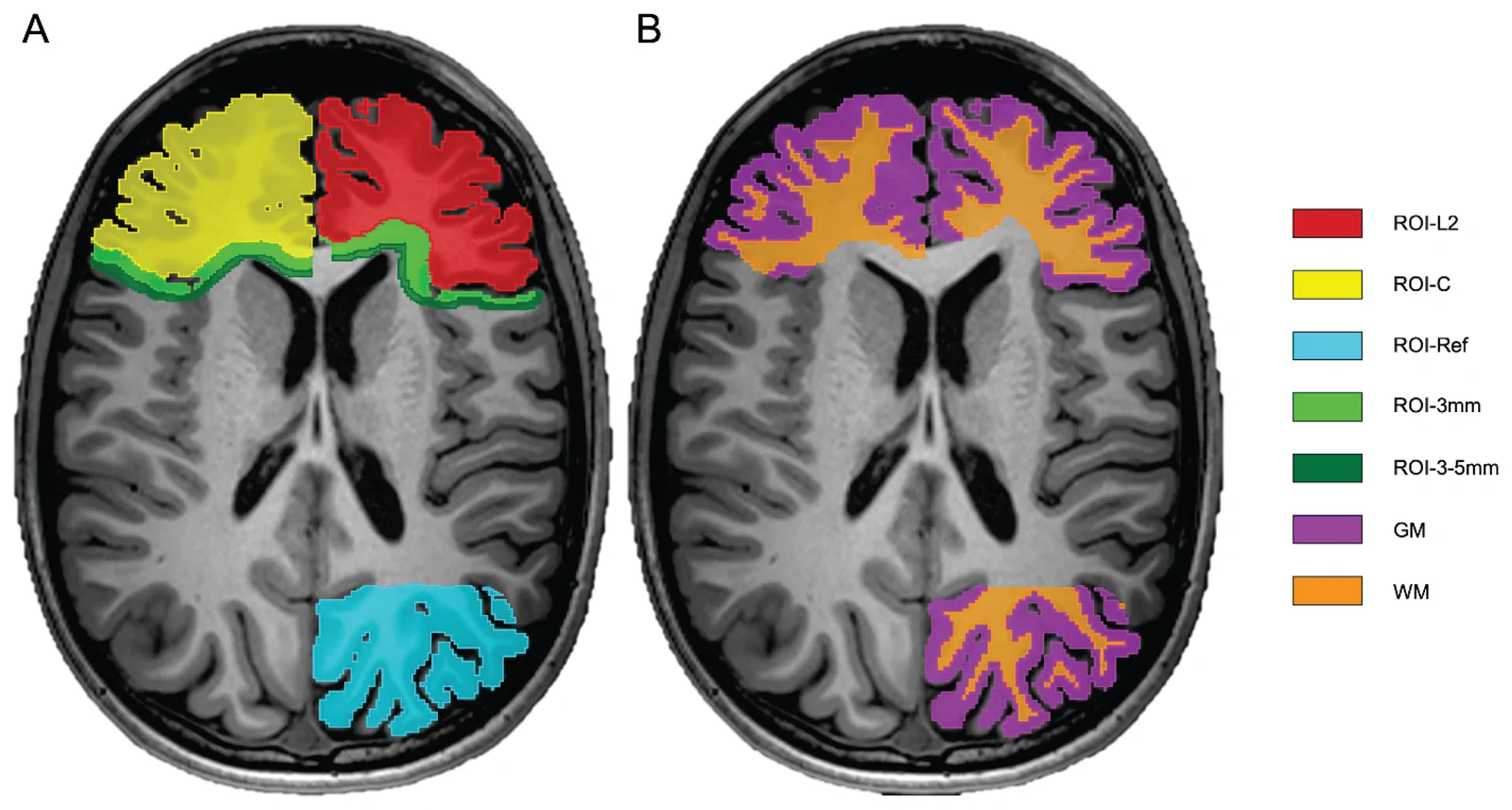
Patient S04, FCD 1A in the left frontal lobe, T1w. (**A**) 5 regions of interest – lesion 2 (red), contralateral/healthy region (yellow), ipsilateral/reference region (blue), perilesional tissue in 3 mm (light green) and 3–5 mm (dark green). (**B**) Example of automatic segmentation of regions into grey (GM, purple) and white matter (WM, orange).

Five regions of interest (ROIs) were defined in this study (Fig. 1):

1. ROI-L2 (lesion 2): final lesion mask derived from expert contextual assessment.
2. ROI-3: 3-mm perilesional band surrounding ROI-L2.
3. ROI-3–5: outer 2-mm band immediately beyond ROI-3.
4. ROI-C (contralateral region): anatomically mirrored region in the healthy hemisphere.
5. ROI-Ref (reference region): ipsilateral region distal to the lesion with preserved grey-white differentiation.

Each ROI was automatically segmented into grey and white matter. Resected tissue was manually delineated by comparing pre- and post-surgical MRIs.

To obtain inter-reader variability, lesion 1 (ROI-L1) was defined as a feature-focused delineation based on a first independent reading performed by a radiologist with expertise in epilepsy imaging (NP), who analyzed the epileptogenic lesions in each structural sequence, without prior knowledge of the lesion’s location or the epileptogenic zone. The analysis concentrated on regions exhibiting the most striking MRI features of FCD.

Delineation of ROI-L2 was performed as a second reading by an experienced pediatrics neuroradiologist (ZH) specializing in epilepsy patients, blinded to ROIs-L1 and post-surgical MRIs but familiar with the site of FCD. The analysis incorporated also visible changes in white matter volume, subtle changes of cortical and white matter signal intensity, took into account changes in gyrification and contextual knowledge. Manual segmentation was done in T1w sequence with slight correction to T2w and FLAIR sequences which brought auxiliary information.

To assess perilesional signal characteristics, SI values were additionally analyzed in two concentric perilesional bands (0–3 mm and 3–5 mm from the lesion border) and compared with the corresponding mirrored regions in the contralateral hemisphere.

The ipsilateral reference region ROI-Ref was included to verify that signal characteristics of the contralateral mirrored region ROI-C represented normal tissue; therefore, SI values in ROI-Ref were systematically compared with ROI-C across all sequences and tissue compartments.

The resection cavity was delineated on the early postoperative T1-weighted MRI by comparison with the preoperative T1-weighted scan. The postoperative image was co-registered to the preoperative T1w space, and a binary cavity mask was manually segmented in 3D Slicer and used for overlap analyses.

Visual conspicuity of lesions on qT1 vs. T1w was independently evaluated for each patient by an experienced neuroradiologist (ZH) using an ordinal scale (0–2) for lesion visibility (0=not visible, 1=visible, 2=clearly visible) and for overall comparative conspicuity between qT1 and T1w (0=worse, 1=equal, 2=better); results were analyzed descriptively at the group level.

### Statistical analysis

Signal-intensity (SI) values and ROI volumes were extracted in 3D Slicer using the built-in Segment Statistics module [42]. For each ROI and sequence, SI was defined as the median voxel value calculated for the whole ROI as well as separately for voxels segmented as grey or white matter. SI contrasts were computed as the difference between median SI values of the compared ROIs.

Primary comparisons were conducted within each patient using paired intra-subject analyses to minimize inter-scan variability, and group-level analyses evaluated the distribution of these within-subject contrasts across the cohort and, secondarily, between FCD subtypes. Analyses were hypothesis-driven and organized into a limited number of pre-specified comparison families aligned with the study aims. Paired tests (*t*-test or non-parametric equivalents, as appropriate) were used for ROI comparisons, and linear mixed-effects models were applied for multilevel analyses; secondary subtype-related analyses were interpreted as exploratory.

Spatial agreement between lesion masks (ROI-L1, ROI-L2) and the resection cavity was assessed using the Dice coefficient. Analyses were performed for whole ROIs and separately for grey- and white-matter segments. Statistical significance was set at p<0.05. All analyses were performed in R (version 4.3.3).

## Results

### Clinical cohort

Thirteen pediatric patients (age range 17 months – 17 years; mean ± SD = 6.7±4.7 years; 69 % female) met inclusion criteria. Seven patients had FCD type I and six FCD type II (reclassified according to the current ILAE classification).

Surgical approaches included tailored focal resections (n=5), lobar resections (n=3), multilobar resections (n=3), and functional hemispherotomy (n=2). Three patients underwent invasive monitoring prior to surgery. Median epilepsy duration was 2.2 years (IQR 3.4), with seizure frequency ranging from several per week to dozens per day. The median interval between MRI and surgery was 3 months (IQR 2.5).

Postoperative seizure outcomes were favorable in most patients: 10 of 13 achieved ILAE class 1 at ≥12-month follow-up. Three patients (S04, S05, S09) remained in ILAE class 4–5 following deliberate partial resections; patient S04 underwent multiple resections and had the poorest outcome in the cohort. Detailed demographic and clinical data are provided in Supplementary Table 2.

### 3.2. Larger expert-contextual lesion masks better align with resection cavity

**Fig. 2:**
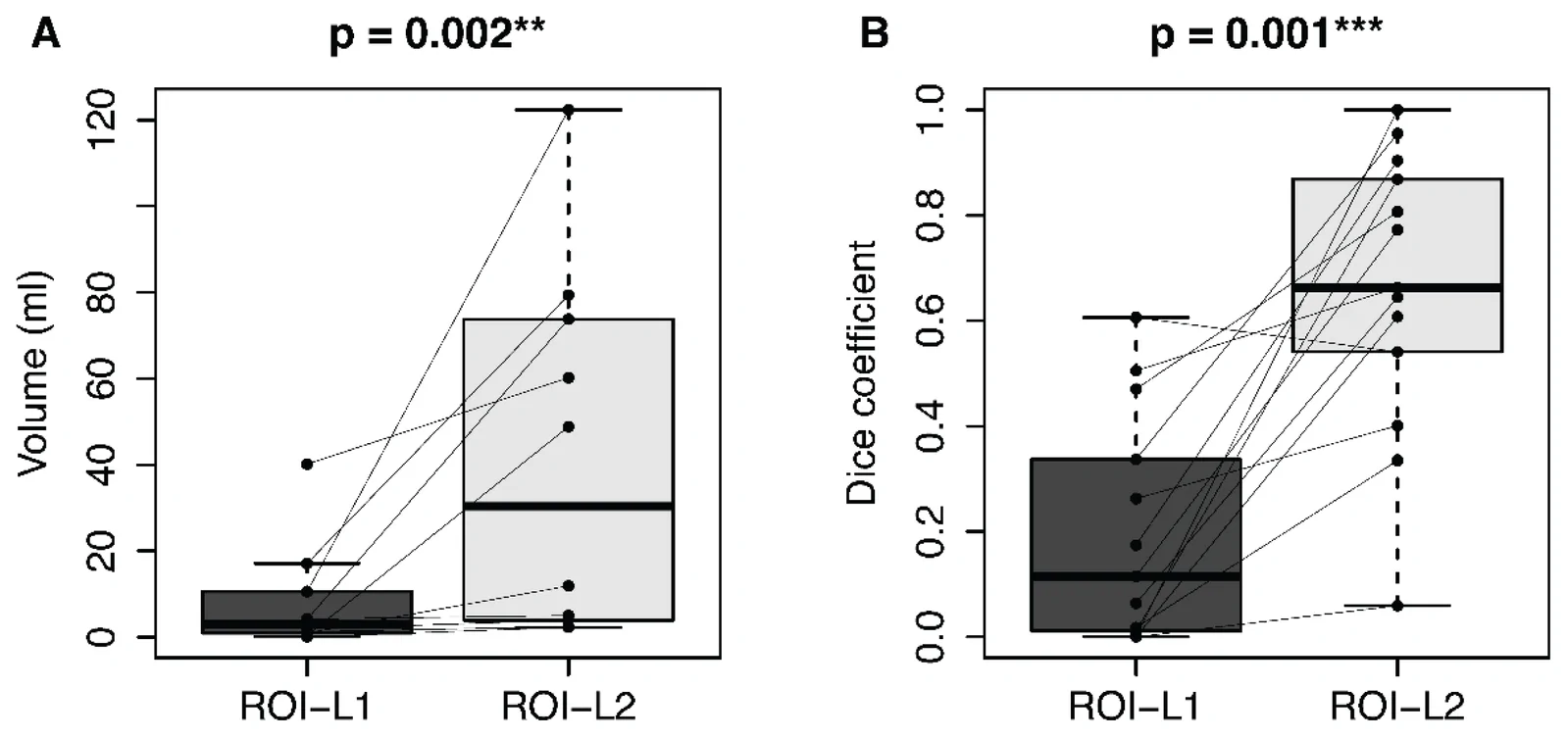
(**A**) Comparison of lesion volumes. Volume of ROIs-L2 is significantly larger than ROIs-L1. ROI-L1 is a subset of ROI-L2 in most cases (Figs 3, 4, 10). (**B**) Spatial correspondence of lesion delineations with the surgically resected tissue. Significantly higher Dice similarity coefficients for ROIs-L2.

**Fig. 3:**
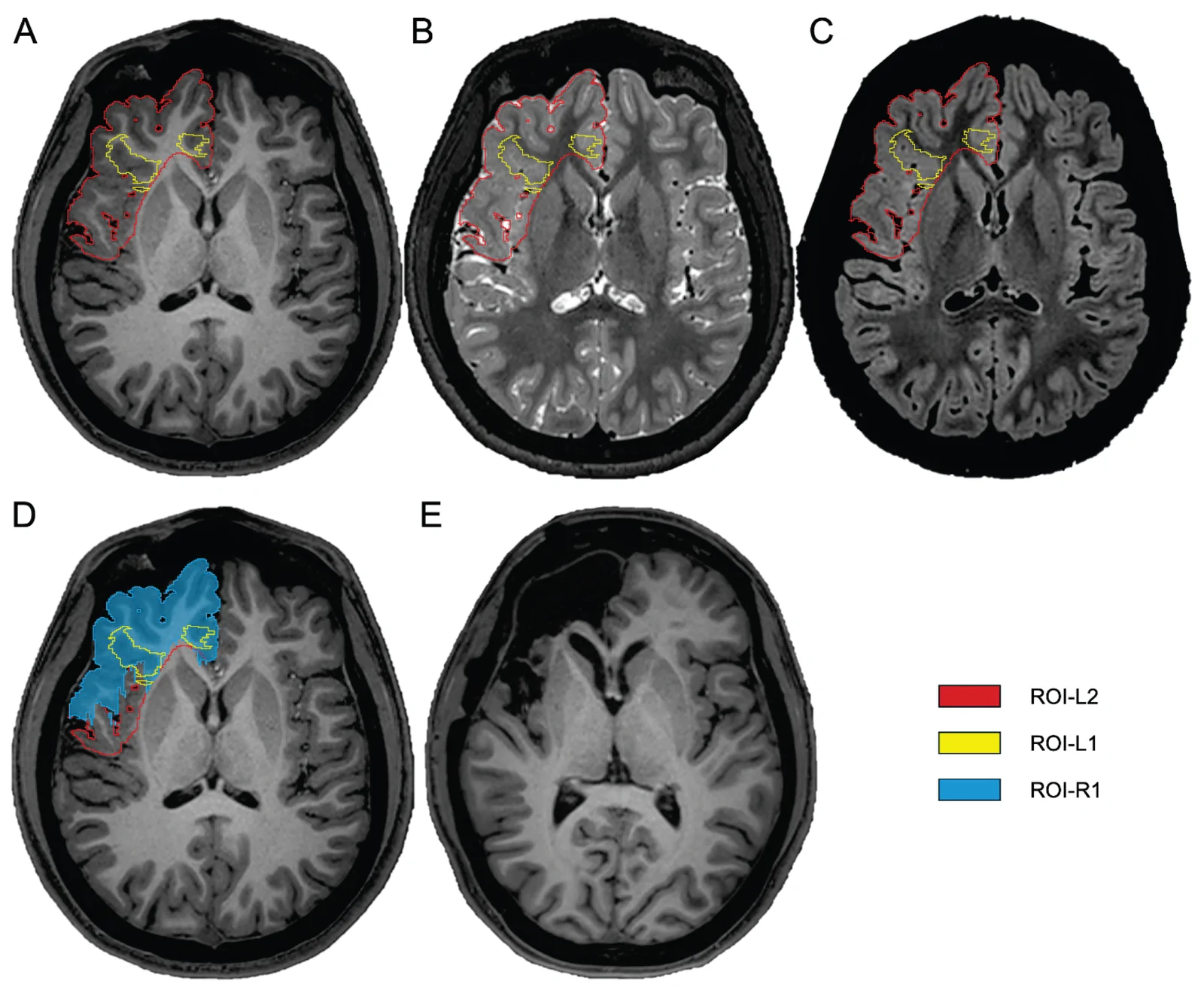
Patient S07, FCD 1A in the right frontal lobe, insula and frontal operculum. (**A**) T1w. Pronounced volume disproportion of ROI-L2 vs. ROI-L1, which was targeted at the most striking FCD features (in yellow). Notice volume loss of white matter in right frontal lobe, frontal operculum and insula, accompanied by subtle signal changes (in red). Correlation in T2w and FLAIR image (**B, C**). (**D**) Resection mask in blue displays quite good overlap with ROI-L2. (**E**) T1w. Follow-up 1 year after surgery.

**Fig. 4:**
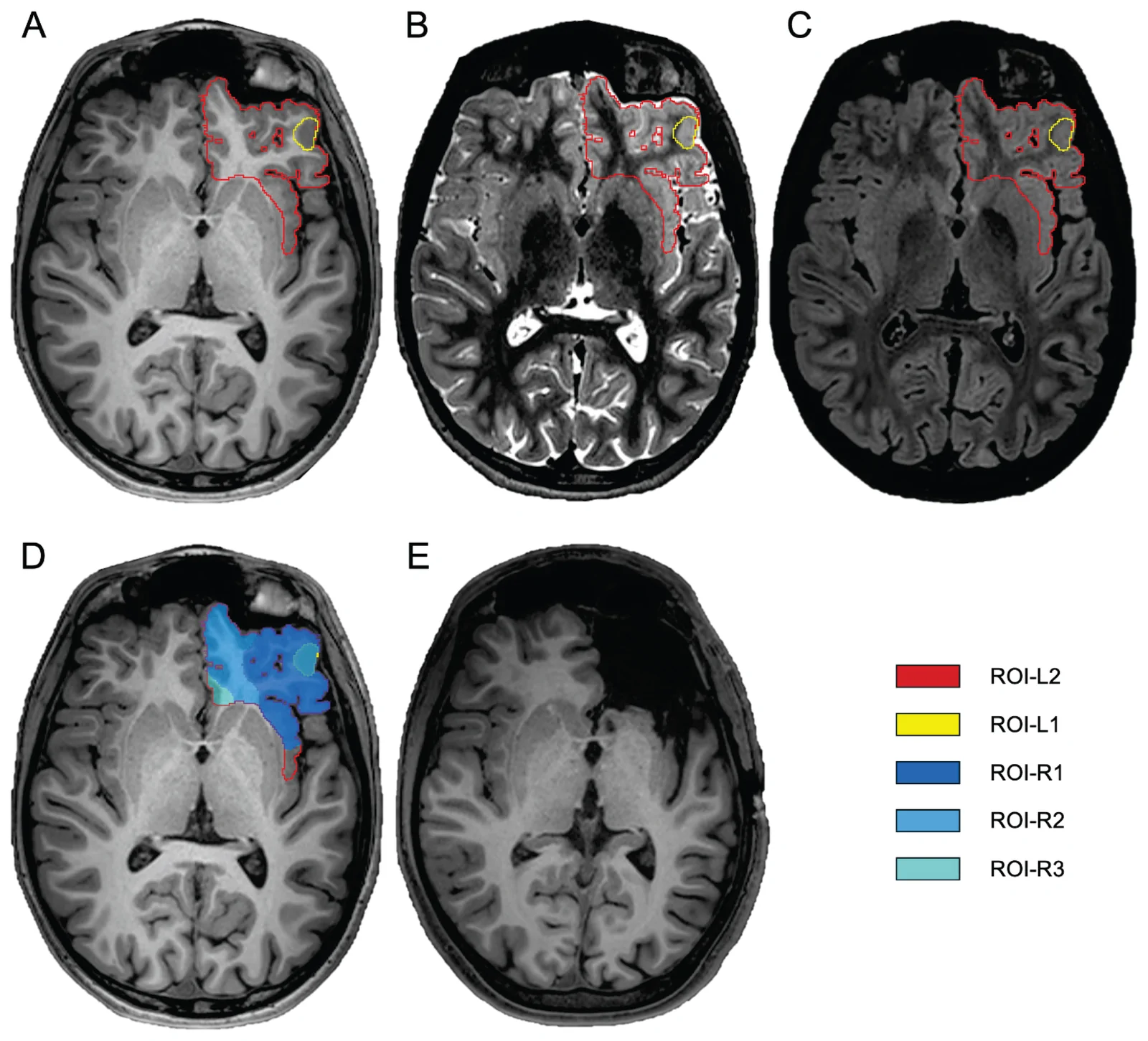
Patient S04, FCD 1A in the left frontal lobe and insula. (**A**) T1w. Pronounced volume disproportion of ROI-L2 vs. ROI-L1, which was targeted at the most striking FCD features (in yellow). Notice very subtle volume loss of white matter in left frontal lobe, accompanied by subtle signal changes (in red). Correlation in T2w and FLAIR image (**B, C**). (**D**) Resection masks in blue – repeated surgeries gradually extended over the frontal lobe and insula reaching very good overlap with ROI-L2. (**E**) T1w. Follow-up 9 months after the last surgery. ROI-R1, ROI-R2, ROI-R3 – resection masks of the 1^st^, 2^nd^ and 3^rd^ surgery

A comparison of lesion volumes showed that ROI-L2 was consistently and substantially larger than ROI-L1 (median 18.2 cm^3^ vs. 3.0 cm^3^; p=0.002). Nearly all paired observations demonstrated this pattern, indicating that the expert-contextual delineation (ROI-L2) encompassed a broader extent of abnormal tissue than the feature-focused mask (ROI-L1) (Figs 2–4).

Spatial correspondence with the surgically resected tissue differed markedly between the two approaches. ROI-L2 achieved significantly higher Dice similarity coefficients (median 0.66) compared with ROI-L1 (median 0.11; p=0.001), demonstrating substantially greater overlap with the resection cavity. This effect was consistent across subjects (Figs 2–4).

Together, these findings indicate that the expert-contextual delineation (ROI-L2) captures a substantially larger volume and more closely aligns with the surgically removed tissue than the feature-focused delineation (ROI-L1).

### Ipsilateral reference region is comparable to healthy brain

Signal intensities in the ipsilateral reference region ROI-Ref did not differ from those in the contralateral region ROI-C across any MRI sequence or tissue class (all p>0.41), with near-identical median SI distributions (Fig. S1, Table S3). The absence of detectable SI differences remained consistent when analyzing grey and white matter separately. These findings confirm that the reference region closely matches the contralateral healthy tissue and does not provide additional information beyond ROI-C. Consequently, subsequent analyses focus on comparisons with the contralateral region.

### Signal-intensity differences between lesions and healthy tissue

#### Feature-focused lesion mask (ROI-L1) demonstrates increased signal intensity relative to contralateral healthy tissue

**Fig. 5:**
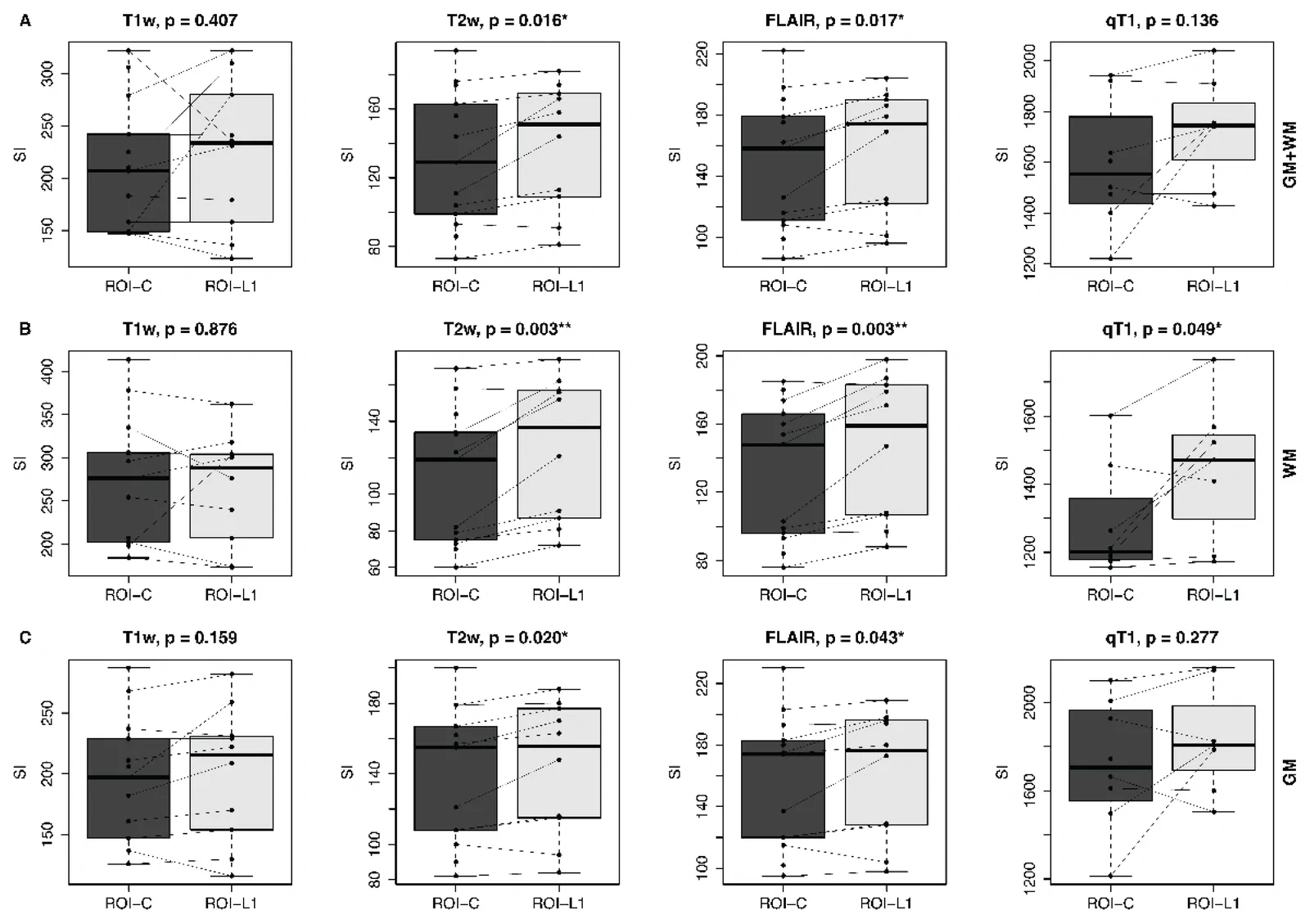
Difference in signal intensities ROI-L1 vs. ROI-C. (**A**) Whole volume analysis. (**B**) WM-only analysis. (**C**) GM-only analysis. */** – degree of significance, p value of 2 or 3 decimals.

ROI-L1 showed consistently higher SI than the contralateral region across the cohort. The strongest differences were observed on T2w and FLAIR images, where both mean and median SI were significantly elevated (p<0.017; Fig. 5). These effects were preserved in both GM- and WM-only analyses, with the WM-only comparison showing the most stable and least variable contrast (p=0.003). For qT1, significant differences between ROI-L1 and ROI-C emerged only in the WM-only analysis (p<0.05).

#### White matter signal abnormality is the distinctive factor for expert-contextual lesion masks

**Fig. 6:**
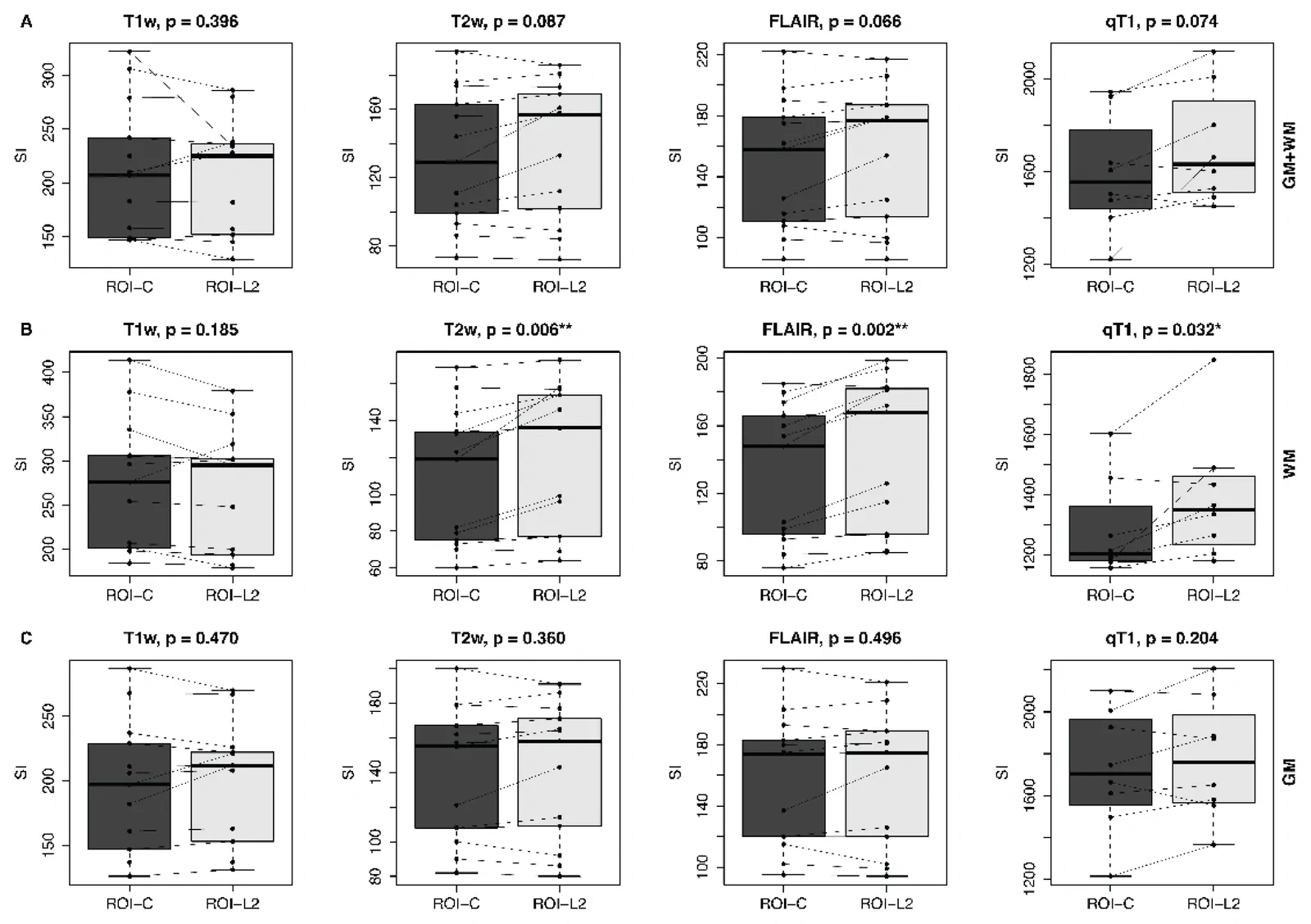
Difference in signal intensities ROI-L2 vs. ROI-C. (**A**) Whole volume analysis. (**B**) WM-only analysis. (**C**) GM-only analysis. */** – degree of significance, p value of 2 or 3 decimals.

Compared with ROI-L1, the expert-contextual mask (ROI-L2) showed weaker and less consistent SI deviations from healthy tissue. In whole-volume analyses, none of the sequences reached statistical significance, although T2w, FLAIR, and qT1 demonstrated trends toward higher SI (p=0.06–0.09). In WM-only analyses, SI was significantly elevated in ROI-L2 for both T2w (p<0.007) and FLAIR (p<0.002), whereas GM-only analyses showed no significant differences. For qT1, significant WM-only differences were again observed (p=0.03) (Fig. 6).

#### Grey matter signal abnormality is the distinctive factor between lesion masks

**Fig. 7:**
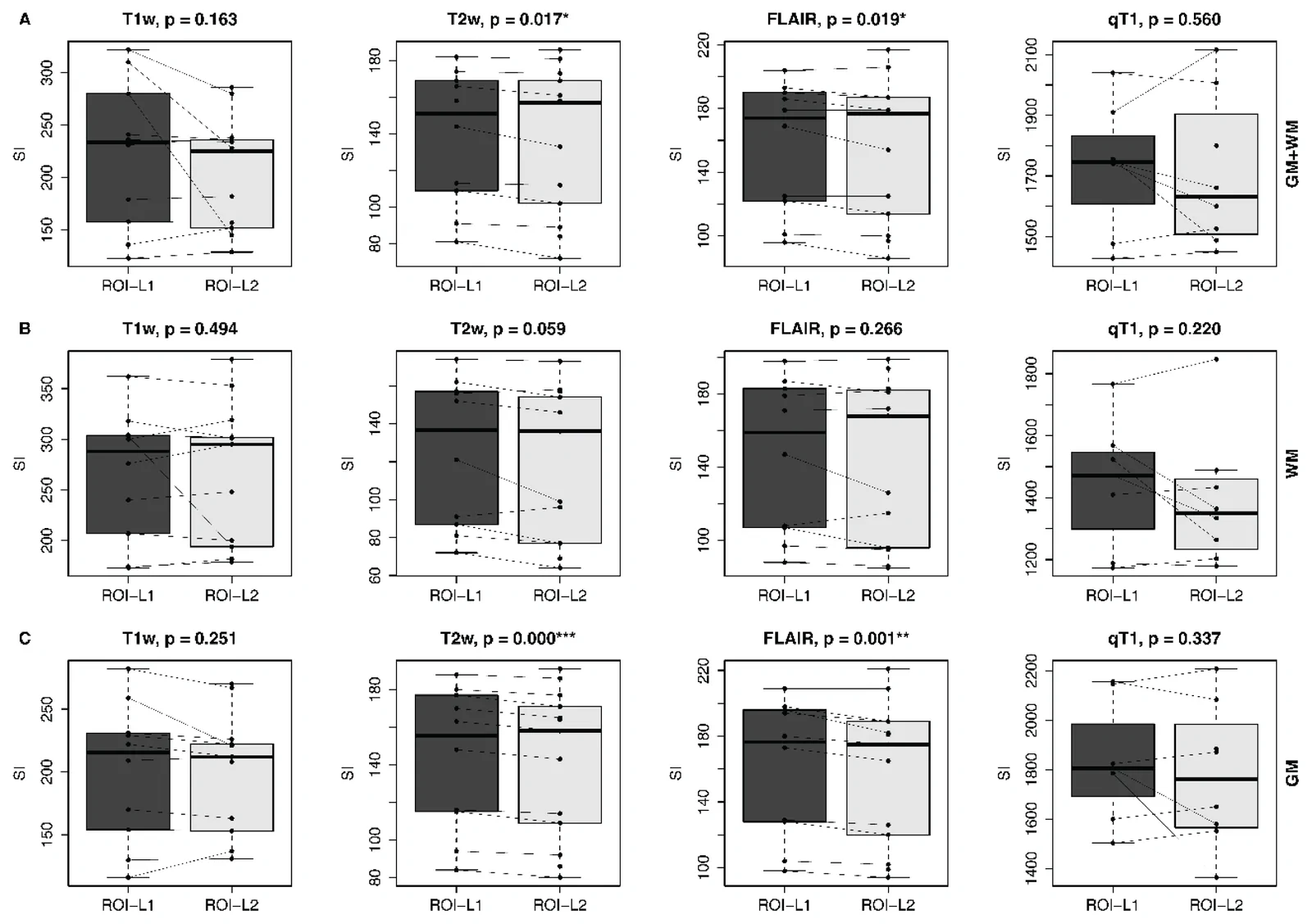
Difference in signal intensities ROI-L1 vs. ROI-L2. (**A**) Whole volume analysis. (**B**) WM-only analysis. (**C**) GM-only analysis. */**/*** – degree of significance, p value of 2, 3 or 4 decimals.

Direct comparison of the two lesion masks revealed modest but sequence-dependent SI differences. In whole-volume analyses, ROI-L1 and ROI-L2 differed significantly only on T2w and FLAIR, with small effect sizes. WM-only analyses did not show significant differences (trend for T2w: p=0.06), whereas GM-only analyses demonstrated marked differences on both T2w (p<0.0001) and FLAIR (p=0.0014). These findings indicate that the most pronounced distinction between ROI-L1 and ROI-L2 arises from grey-matter SI abnormalities (Fig. 7).

Across sequences, T2w and FLAIR provided the clearest lesion-lesion contrast, while T1w and qT1 showed weaker or absent differences.

#### Better conspicuity of FCD type II at the white-matter level

Across most comparisons, SI contrasts showed only limited differences between FCD type I and II. For the ROI-L1 vs. ROI-C and ROI-L1 vs. ROI-L2 metrics, contrasts tended to be slightly higher in FCD II, but none reached statistical significance.

**Fig. 8:**
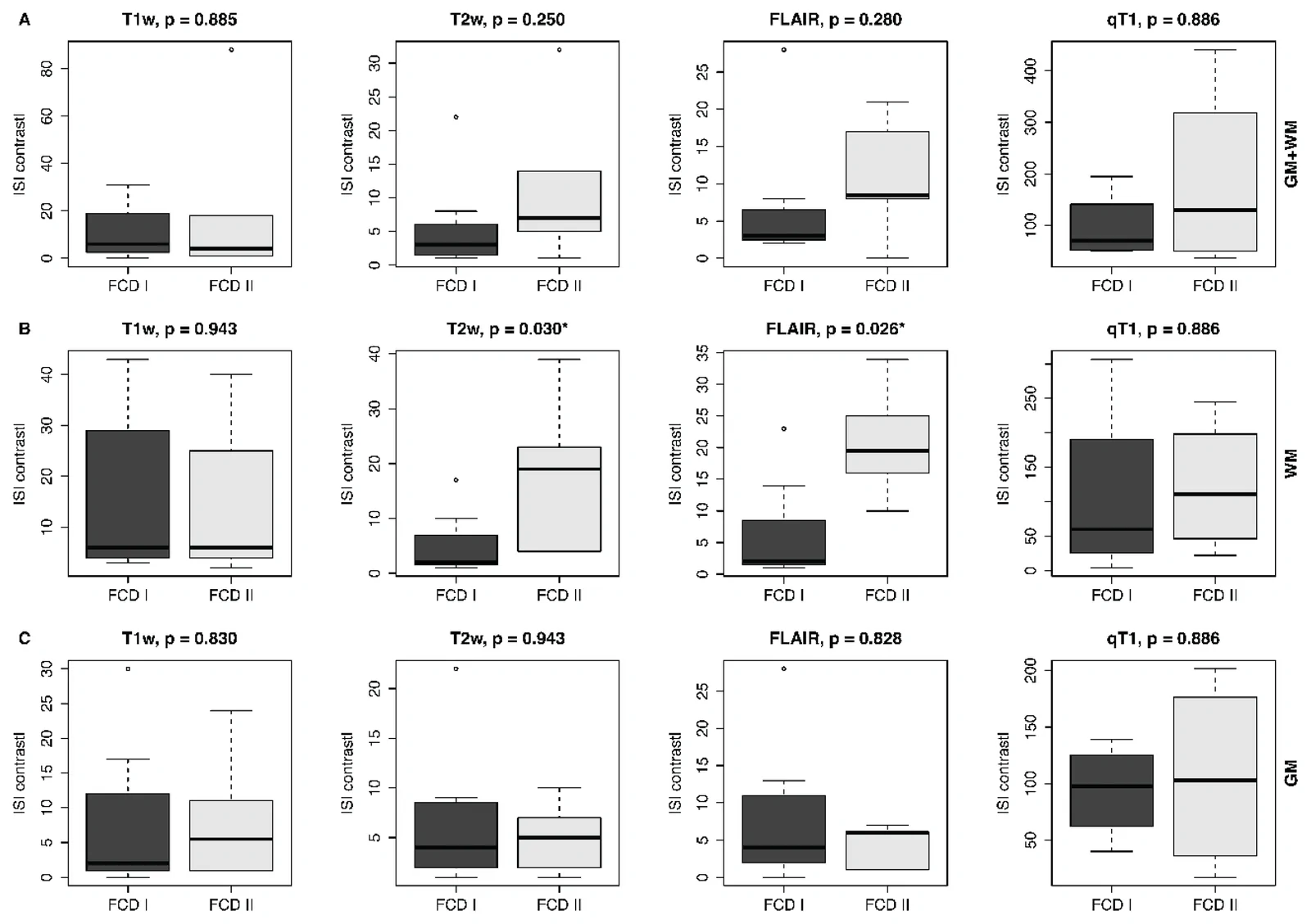
Difference in signal intensities ROI-L2 vs. ROI-C between FCD type I and II. (**A**) Whole volume analysis. (**B**) WM-only analysis. (**C**) GM-only analysis. * – degree of significance, p value of 2 decimals.

**Fig. 9:**
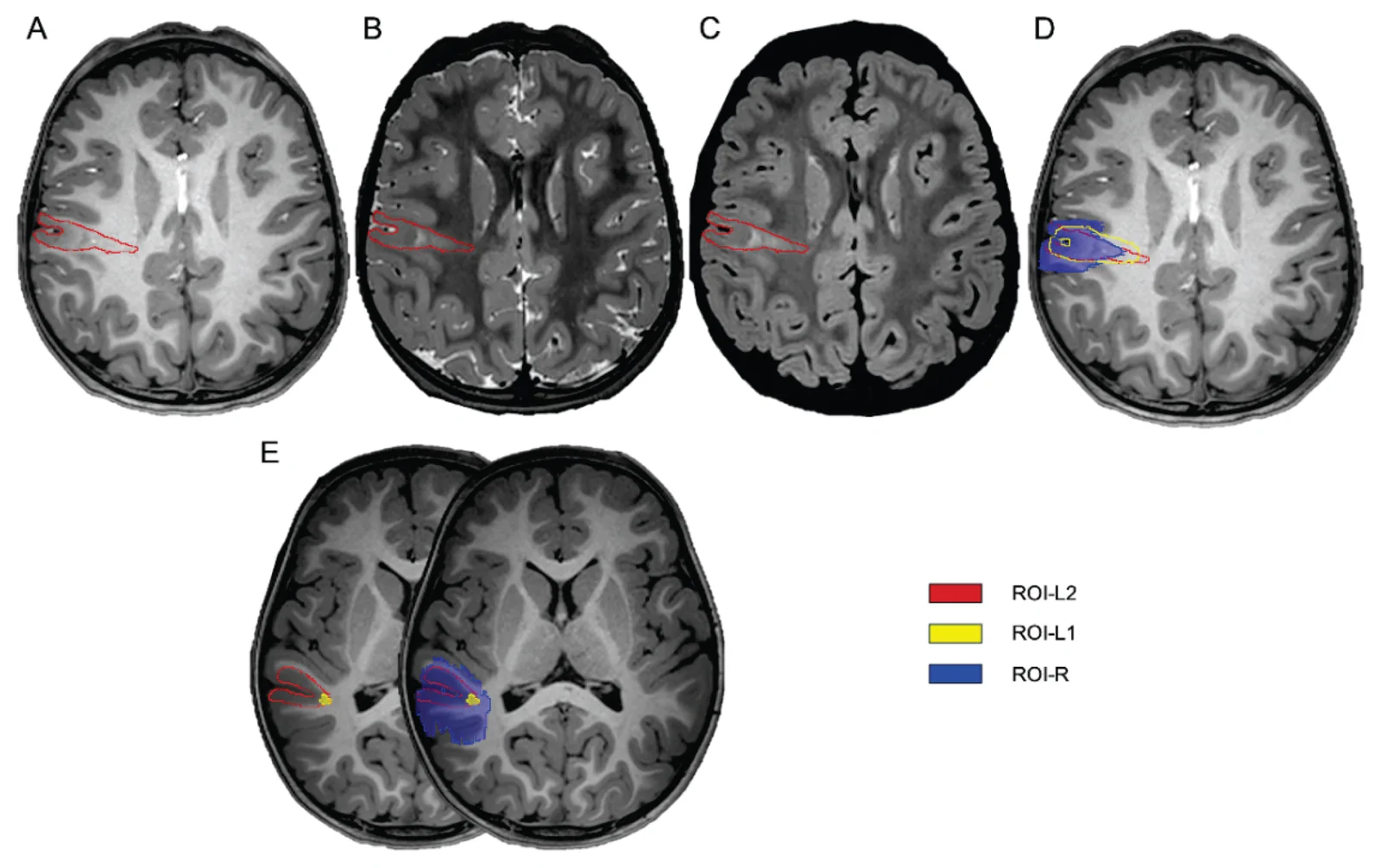
Patients S08 and S11, FCD 2B. (**A**) Patient S08. T1w. FCD 2B with classical features – thickened cortex with signal changes, maximum changes at the bottom of sulcus, conspicuous signal changes of subcortical white matter and transmantle sign. Correlation in T2w and FLAIR image (**B, C**). (**D**) Resection mask in blue – quite good overlap with ROI-L1 and ROI-L2. (**E**) Patient S11. T1w. Example of very poor correlation of ROI-L1 and ROI-L2. Notice that resection area is 2–3 times bigger than ROI-L2. ROI-L1 – yellow, ROI-L2 – red, ROI-R – resection mask, blue.

More distinct subtype effects were observed for the ROI-L2 vs. ROI-C contrast in the WM-only analyses. Here, FLAIR (p=0.026) and T2w (p=0.031) demonstrated significantly greater SI contrast in FCD II compared with FCD I, while qT1 showed a similar trend but with high variability. T1w provided minimal discriminatory value across subregions (Fig. 8, Fig. 9).

### Characteristics of perilesional tissue

#### Lesion core shows a sharp signal transition at the lesion boundary

**Fig. 10:**
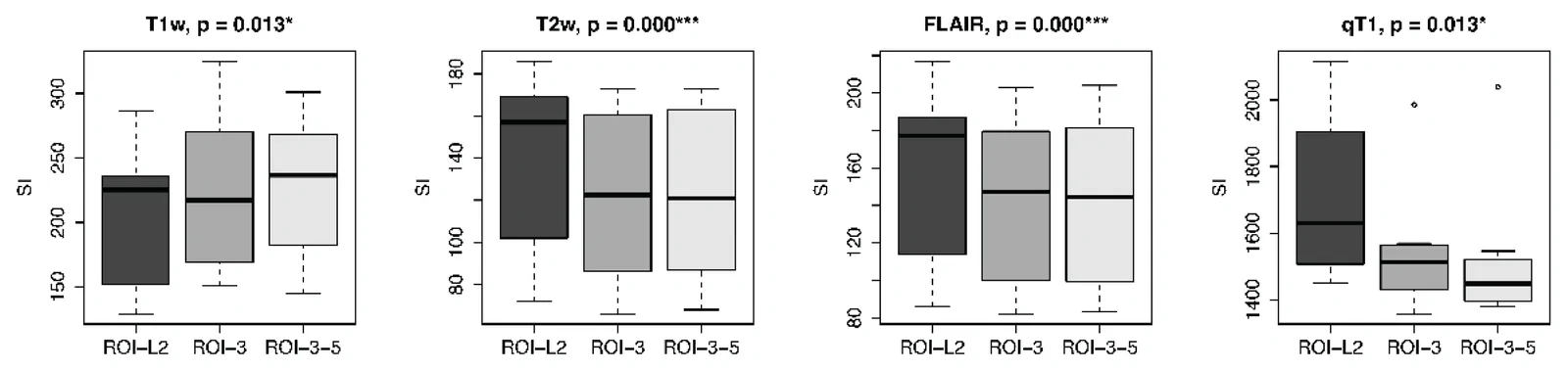
Lesion core vs. perilesional zones. Differences were significant across all sequences in the linear mixed-effects model. The most prominent SI change occurred between the lesion and ROI-3 – at lesion boundary. */*** – degree of significance, p value of 2 or 4 decimals.

Across all MRI sequences, SI differed significantly between ROI-L2 and the immediate 3-mm perilesional zone (ROI-3), with the largest effects on T2w and FLAIR (linear mixed-effects model; Fig. 10). SI values progressively approached normal tissue with increasing distance from the lesion. No significant differences were observed between ROI-3 and ROI-3–5 in any sequence, indicating that the principal SI change occurs at the lesion boundary. T1w showed minimal gradients.

The same analysis performed in the contralateral hemisphere did not demonstrate any significant SI gradients between ROI-C and its adjacent zones across sequences, confirming that the observed ipsilateral gradients are pathology-specific rather than reflecting normal anatomical variation.

#### Perilesional tissue shows signal intensity comparable to healthy brain

**Fig. 11:**
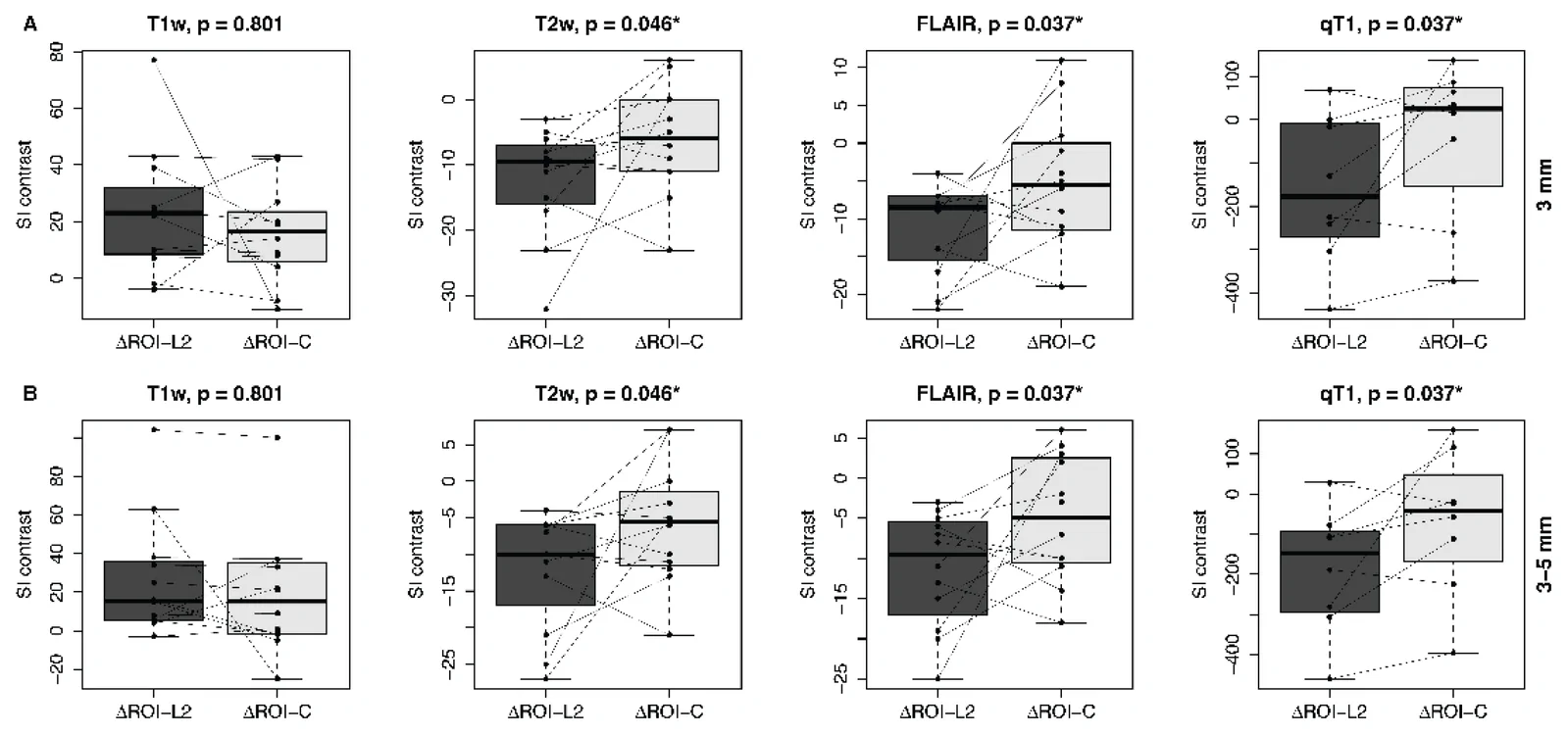
Similarity of perilesional tissue to lesion core vs. healthy tissue. (**A**) 3-mm perilesional band surrounding ROI-L2 (ROI-3). (**B**) Outer 2-mm band immediately beyond ROI-3 (ROI-3–5). In T2w, FLAIR, and qT1 sequences, a significantly larger SI contrast between lesion and perilesional zones was observed than in contralateral region and the same zones. * – degree of significance, p value of 2 decimals.

For T2w, FLAIR and qT1, SI contrast between ROI-L2 and perilesional tissue was significantly greater than the contrast between perilesional tissue and ROI-C (p≈0.037–0.046; Fig. 11). The perilesional-to-contralateral contrast approached zero across sequences, indicating that perilesional tissue more closely resembles healthy brain than the lesion core. No such effects were observed on T1w.

#### Perilesional tissue does not significantly differ among FCD types

**Fig. 12:**
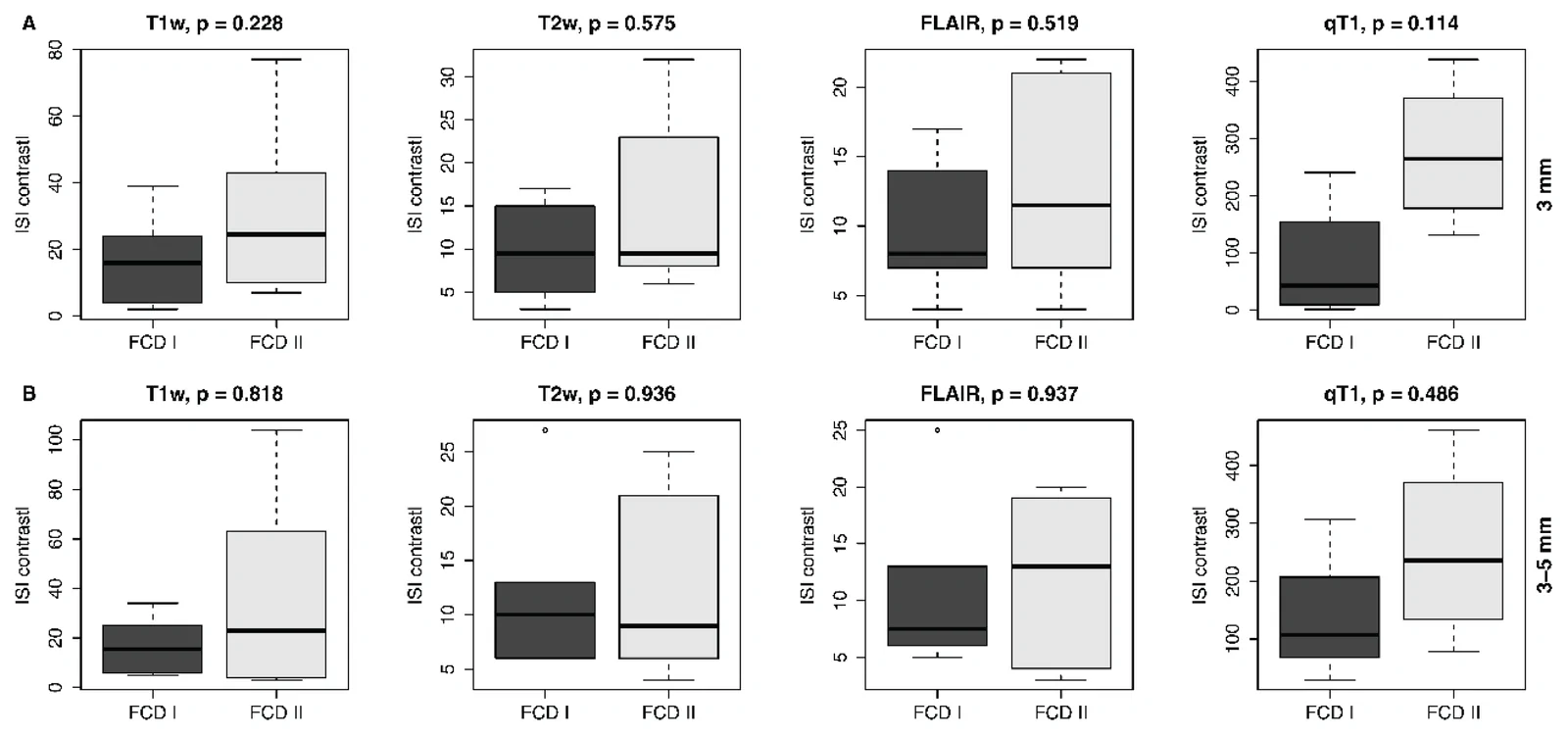
Differences between FCD type I and type II in perilesional tissue. (**A**) 3-mm perilesional band surrounding ROI-L2 (ROI-3). (**B**) Outer 2-mm band immediately beyond ROI-3 (ROI-3–5). SI contrast between ROI-L2 and perilesional tissue does not significantly differ between FCD type I and II.

SI contrast between ROI-L2 and perilesional tissue was consistently larger in FCD type II than in type I across sequences and zones, although none of the comparisons reached statistical significance (all p>0.1). The greatest subtype separation was observed on qT1, where median contrasts were nearly doubled in FCD II. T1w showed similar but smaller trends, while T2w and FLAIR displayed only subtle differences (Fig. 12).

### Limited added value of qT1 at the white-matter level

**Fig. 13:**
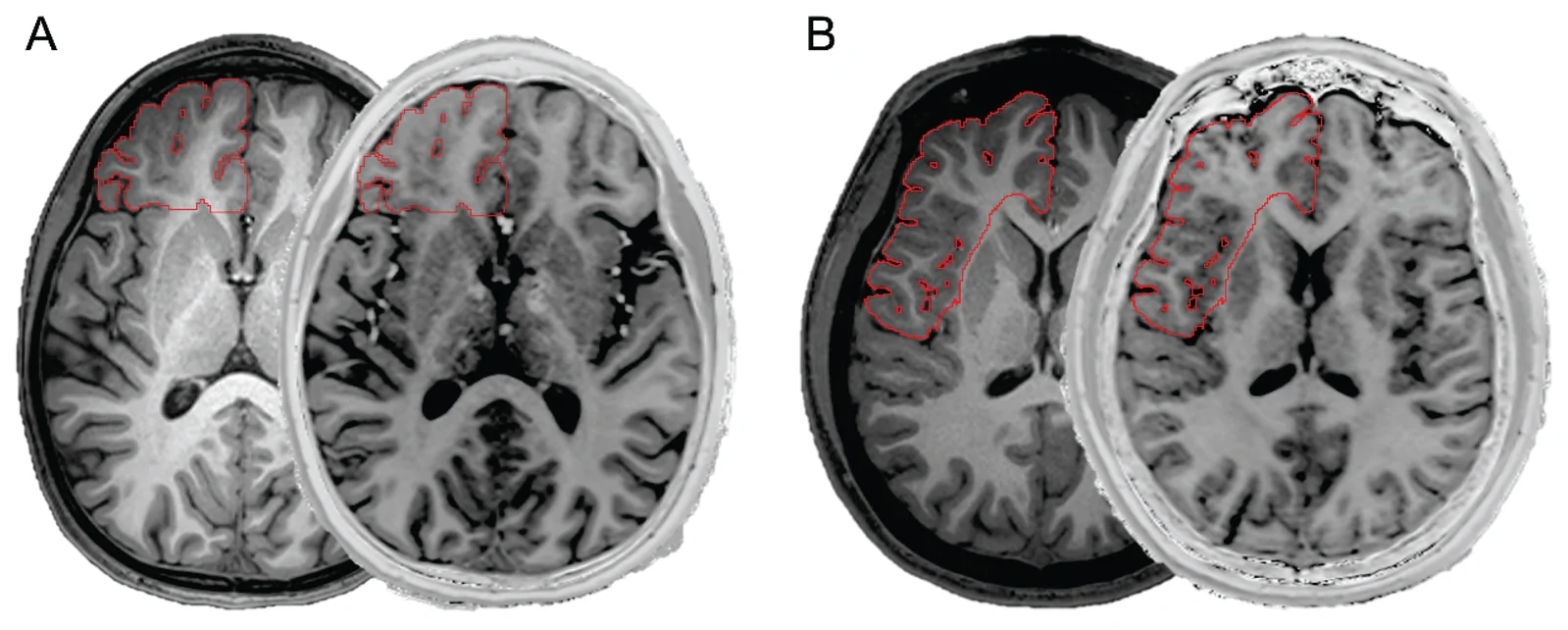
Patients S01 and S07, FCD 1A. (**A**) T1w, qT1. Visual assessment of lesion conspicuity. Blurring and signal changes can be better appreciated on qT1 (right). (**B**) T1w, qT1. Visual assessment of lesion conspicuity. Blurring and signal changes can be better appreciated on T1w (left).

qT1 imaging was available in 8 of 13 patients (balanced across FCD types I and II). Visual assessment showed comparable lesion conspicuity between qT1 and T1w in half of the cases, with qT1 providing better visibility in two patients and worse visibility in two others (Fig. 13).

In quantitative analyses, significant SI differences on qT1 were present only in WM-only comparisons between the lesion masks (ROI-L1 or ROI-L2) and the contralateral region (p<0.05 and p=0.03, respectively). qT1 also differentiated the lesion from perilesional tissue more effectively than T1w (p=0.037) and showed greater perilesional contrast in patients with FCD type II. No comparable differences were observed on T1w.

## Discussion

Complete resection of dysplastic tissue is critical for achieving seizure freedom in children with FCD [43,44]. However, many lesions, especially type I, remain subtle or MRI-negative, leading to inter-reader variability and reliance on expert judgement [11,15,45]. We therefore examined how lesion delineation strategies and quantitative SI metrics relate to surgical resections and tissue compartments to clarify which imaging features best define the epileptogenic lesion.

In line with prior work, our results showed that T2-weighted and FLAIR imaging provided the strongest signal-intensity differences between FCD lesions and healthy tissue, consistent with their established sensitivity to cortical and subcortical abnormalities in FCD, particularly type II [15,38,45]. Although T1-weighted imaging remains the preferred sequence for estimating lesion extent in clinical practice and forms the backbone of HARNESS-MRI protocols [40], its quantitative contrast between dysplastic and normal tissue was comparatively weak. Quantitative T1 mapping demonstrated higher sensitivity than T1w in white-matter–only analyses, a finding that may reflect underlying microstructural alterations, yet qT1 did not outperform T2w/FLAIR nor differentiate lesion definitions robustly [26,46]. Together, these results underscore the dominant role of T2w and FLAIR contrast in detecting lesional tissue, while also suggesting a complementary, although limited, role for qT1 in characterizing white-matter abnormalities.

Our findings demonstrated that GM abnormalities were primarily responsible for the strongest differences between the two lesion masks, consistent with the cortical origin and histopathological characteristics of FCD [2,17,39]. In contrast, WM abnormalities remained detectable across both lesion definitions and MRI sequences, suggesting that WM alterations reflect broader network-level or radial pathology extending beyond the cortical ribbon [29,38,47]. These observations align with diffusion MRI studies showing widespread microstructural abnormalities in WM tracts associated with FCD, even when cortical changes are subtle. Together, the differential behavior of GM and WM underscores the value of assessing tissue compartments separately rather than relying solely on whole-volume masks.

The two lesion delineation strategies captured complementary aspects of the epileptogenic zone. The feature-focused mask (ROI-L1) showed stronger and more consistent signal-intensity differences relative to healthy tissue, indicating that it primarily reflects the most conspicuous dysplastic features. However, ROI-L1 exhibited limited spatial correspondence with surgically resected tissue. In contrast, the expert-contextual mask (ROI-L2) was substantially larger and more heterogeneous yet demonstrated markedly better overlap with the resection cavity, highlighting the importance of integrating subtle white-matter abnormalities, gyral morphology and contextual anatomical cues, elements known to influence expert detection of FCD [10,40,45]. Resection cavity overlap should be interpreted as concordance with the clinically targeted resection rather than a direct ground-truth measure of the epileptogenic zone, which is shaped by multimodal presurgical evaluation and surgical feasibility. Moreover, expert delineations may inform surgical decision-making, so overlap metrics reflect agreement with real-world practice rather than an independent validation standard. This divergence between quantitative contrast and surgical relevance suggests that high SI differences alone are insufficient to define the true extent of epileptogenic tissue. It also carries implications for automated detection methods, which often prioritize voxel-wise intensity abnormalities and may therefore fail to capture the broader expert-derived pattern that underlies clinically meaningful lesion delineation [34,48].

Contrary to our initial hypothesis, perilesional tissue exhibited signal-intensity values much closer to contralateral healthy brain than to the lesion core across T2w, FLAIR and qT1 sequences. This pattern suggests that the dysplastic abnormality is sharply confined to the lesional zone, with minimal measurable extension into the immediately adjacent cortex or white matter. Prior imaging and histopathological studies similarly describe relatively abrupt transitions between dysplastic and non-dysplastic tissue in many cases of FCD, particularly type II [49,50]. The absence of corresponding gradients in the contralateral hemisphere further supports the specificity of this effect. From a radiological perspective, these findings underscore that SI-based perilesional abnormalities may not reliably indicate extended dysplastic tissue and should be interpreted cautiously when estimating lesion margins or surgical boundaries.

Signal-intensity contrasts were generally greater in FCD type II than in type I, particularly in white-matter-only analyses of T2w and FLAIR sequences, consistent with the more conspicuous imaging phenotype typically associated with type II lesions [2,17,38]. qT1 demonstrated similar subtype trends but with substantial variability, reflecting heterogeneous microstructural features across dysplasia types [16,46,49]. Overall, the limited group differences observed in quantitative SI analysis highlight the known variability of imaging expression in FCD, especially in type I, where abnormalities are often subtle or non-specific and may not produce robust quantitative signal deviations.

This study has several limitations. First, the modest cohort size reflects the rarity of histologically confirmed pediatric focal cortical dysplasia and our inclusion of protocol-consistent, single-scanner examinations within a single epilepsy surgery program. Although comparable to prior imaging studies, this reduced statistical power and precluded definitive subtype-specific comparisons. Subtype analyses were therefore treated as exploratory and should be interpreted cautiously. Second, automatic GM-WM segmentation may be less reliable at the lesion boundary, where cortical architecture is abnormal and tissue contrast is reduced, potentially affecting boundary-based estimates [51]. Third, quantitative T1 mapping was available only in a subset of patients, which may limit the generalizability of qT1-related findings. Fourth, lesion delineation relied on expert readers and thus incorporates subjectivity; this is inherent to current clinical practice and aligns with evidence of substantial inter-reader variability in FCD detection [14,45]. Finally, the single-center, retrospective design and protocol-specific acquisition parameters may limit generalization to other institutions or scanner platforms.

These constraints are particularly relevant when interpreting discrepancies between voxel-wise intensity metrics and expert-defined lesion extent: intensity-based measures may not fully capture the contextual and morphology-informed features used in clinical reading [14,40,45]. Future work should therefore evaluate multimodal approaches that combine quantitative metrics with contextual and morphological information in larger, multi-center datasets [34,48].

## Conclusions

In summary, our results indicate that expert neuroradiological assessment and quantitative signal-intensity metrics capture complementary aspects of pediatric FCD on MRI. Conventional T1-weighted imaging most closely reflects the visually defined lesion margins used in clinical reading, whereas quantitative measures, particularly qT1 where available, provide a more standardized, intensity-based description and may improve lesion-to-tissue contrast. Importantly, intensity-derived abnormalities were largely confined to the visually defined lesion core and its immediate boundary, with perilesional bands approaching contralateral values. Quantifying lesion-to-tissue gradients therefore provides an objective framework to evaluate whether MRI signal abnormalities extend beyond expert-defined margins. Together, these findings address the unmet need for an objective definition of clinically relevant imaging-defined lesion extent and suggest that future automated or semi-automated tools should integrate quantitative metrics with morphology- and context-informed features to approximate expert judgement more faithfully.

## Supplementary Information









## Abbreviations

DTI: diffusion tensor imaging
FCD: focal cortical dysplasia
FLAIR: fluid-attenuated inversion recovery
GM: grey matter
SEEG: stereoelectroencephalography
SI: signal intensity
SISCOM: Subtraction Ictal SPECT coregistered to MRI
SPECT: single-photon emission computed tomography
qT1: quantitative T1 mapping
T1w: T1-weighted
T2w: T2-weighted
WM: white matter
