# Supplementary material for: Structural and Quantitative MRI Signal Features in Pediatric Focal Cortical Dysplasia: Clinical Relevance
Source: Physiol Res. 2026 Jun 1;75(3):597–612. doi: 10.33549/physiolres.935824 (PMC13557539; doi:10.33549/physiolres.935824)
Supplement: Supplementary file 1 [file 75_597_Suppl_Fig_1.docx]

**Supplementary Fig. 1.** Validation of reference regions. Example of results, GM+WM analysis. (**A**) T1w signal intensities comparison of contralateral vs. reference region, no statistical difference was reached. (**B**) Same analysis for T2w.
