# Supplementary material for: Structural and Quantitative MRI Signal Features in Pediatric Focal Cortical Dysplasia: Clinical Relevance
Source: Physiol Res. 2026 Jun 1;75(3):597–612. doi: 10.33549/physiolres.935824 (PMC13557539; doi:10.33549/physiolres.935824)
Supplement: Supplementary file 2 [file 75_597_Suppl_Table_1.docx]

**Supplementary Table 1.** Acquisition parameters of MRI data (selected from the whole protocol).

| *Sequence* | **Type** | **TR [ms]** | **TE [ms]** | **TI [ms]** | **FA [deg]** | **Averaging** | **Acquisition matrix [mm]** | **Voxel size [mm]** | **Acceleration factor** | **Duration [min:sec]** |
| --- | --- | --- | --- | --- | --- | --- | --- | --- | --- | --- |
| *T1w* | 3D MP-RAGE | 2400 | 2.29 |  | 8 | 1 | 0.8×0.8×0.8 | 0.4×0.4×0.8 | GRAPPA - 2 | 6:47 |
| *T2w* | 3D SPACE | 3200 | 5.72 |  | Variable* | 1.8 | 0.8×0.8×0.8 | 0.4×0.4×0.8 | CAIPIRINHA - 3 | 6:35 |
| *FLAIR* | 3D SPACE | 6000 | 350 | 1900 | Variable* | 1.8 | 1×1×0.9 | 0.5×0.5×0.9 | CAIPIRINHA - 4 | 8:02 |
| *qT1* | Gradient echo | 15 | 2 |  | 4 and 23 | 1 | 1×1×1 | 0.5×0.5×1 | GRAPPA - 2 | 8:45 |

* TSE with variable flip angle of refocusing RF pulses (for detail see Katherine T. Scott. SPACE: An Innovative Solution to Rapid, Low SAR, T2-Weighted Contrast in 3D Spin Echo Imaging. Siemens Medical Solutions Published online 2009). TR (repetition time), TE (echo time), TI (inversion time), FA (flip angle).
