# Supplementary material for: Structural and Quantitative MRI Signal Features in Pediatric Focal Cortical Dysplasia: Clinical Relevance
Source: Physiol Res. 2026 Jun 1;75(3):597–612. doi: 10.33549/physiolres.935824 (PMC13557539; doi:10.33549/physiolres.935824)
Supplement: Supplementary file 3 [file 75_597_Suppl_Table_2.docx]

**Supplementary Table 2.** Demographic, clinical, and surgical characteristics of patients included in the study.

| *Subject* | **Sex** | **Laterality** | **Types of seizures** | **Seizure frequency [D, W, M]** | **FCD type** | **1^st^ seisure**  **[Y, M]** | **Age at MRI**  **[Y, M]** | **Time from MRI to surgery [Mo]** | **Lesion localisation** | **Type of surgery/resection** | **Outcome [ILAE]** | **Outcome [Engel]** | **ECoG** | **videoEEG** | **HD EEG** | **SEEG** | **FDG-PET** | **SPECT** | **fMRI** |
| --- | --- | --- | --- | --- | --- | --- | --- | --- | --- | --- | --- | --- | --- | --- | --- | --- | --- | --- | --- |
| *S01* | F | R | focal, preserved consciousness, epileptic spasms | 3/D | 1A | 4Y 2M | 5Y 3M | 3M | R frontal | multilobar FT | 1 | 1A | yes (+IOM motor fx) | yes | no | no | R temporal | no | no |
| *S02* | F | R | focal, impaired consciousness, tonic-clonic; negative myoclonus, FBTC; ESES | 3/W | 1A | 8Y 3M | 10Y | 5M | R frontal | hemispherotomy | 1 | 1A | no | yes | yes | yes | L central (lightly) | no | yes |
| *S03* | M | R | aura; FBTC | 2-4/M, aura 2/W | 1A | 9Y 10M | 14Y | 1M | R temporal | lobar T | 1 | 1A | yes | yes | yes | no | normal | yes | yes |
| *S04* | M | R | focal, preserved consciousness, arrest, automatisms, tonic, myoclonic | few/W, up to 20-40/D | 1A | 8Y 6M | 10Y | 2M, 7M, 18M | L frontal basal | lobar F + ventral insula (multiple surgeries) | 5 | 4B* | yes | yes | yes | yes, repeated | L orbitofrontal, insula, amygdala | yes | yes |
| *S05* | M | N/A | focal, preserved consciousness, epileptic spasms | 2-4/D | 1A | 0Y 5M | 1Y 5M | 3M | L parietooccipital | multilobar PO (partial) | 4 | 3A | yes | yes | yes | no (age limit) | normal | no | no |
| *S06* | F | N/A | focal, preserved consciousness, epileptic spasms | 5/D | 1A | 0Y 6M | 2Y 8M | 11,5M | R hemisphere | hemispherotomy | 1 | 1A | no | yes | no | no | R hemisphere | no | no |
| *S07* | F | R | focal, preserved consciousness, tonic, automatisms, hyperkinetic | 3-5/D | 1A | 5Y 7M | 17Y | 3M | R frontal | lobar F + ventral insula | 1 | 1A | yes | yes | yes | yes | R frontal mesial | yes | no |
| *S08* | F | R | focal, preserved consciousness, tonic | 1/D | 2B | 0Y 4M | 3Y | 2M | R frontal dorsal | focal F | 1 | 1A | yes (+IOM motor fx) | yes | no | no | R frontal dorsal | no | no |
| *S09* | M | L | focal, preserved consciousness, tonic; FBTC | tens/D | 2A | 0Y 1M | 9Y | 1,5M | L occipital | multilobar PO (partial) | 4 | 3A* | yes (+IOM motor fx) | yes | yes | no | L temporoparietal | no | no |
| *S10* | F | R | focal, preserved consciousness, tonic, myoclonic | few/W | 2B | 3Y 7M | 5Y | 4M | L frontal precentral | focal F | 1 | 1A | yes (+IOM motor fx) | yes | yes | no | L frontal precentral | no | no |
| *S11* | F | R | focal, preserved consciousness, tonic | 3-5/D | 2B | 1Y 6M | 2Y 1M | 9M | R temporoparietal | focal TP (g. supramarginalis) | 1 | 1A | yes (+IOM motor fx) | yes | yes | no | R temoroparietal | no | no |
| *S12* | F | R | focal, impaired consciousness, psychic aura | 1/D | 2A | 2Y 11M | 8Y | 4M | L frontal mesial | focal F | 1 | 1A | yes | yes | yes | no | L frontal mesial | no | no |
| *S13* | F | R | focal, impaired consciousness, tonic, sensoric, hyperkinetic, emotional | 6-7/D | 2B | 0Y 6M | 3Y 8M | 2M | L occipital | focal O | 1 | 1A* | yes | yes | no | no | L occipital | no | no |

Outcome Engel* (preliminary outcome at 1 year after surgery). FBTC (focal-to-bilateral tonic-clonic seizure), ESES (electrical Status Epilepticus in Sleep). F, T, P, O (frontal, temporal, parietal, occipital). EcoG (intraoperative electrocorticography), IOM motor fx (intraoperative monitoring of motor function). HD EEG (high density EEG), SEEG (stereoelectroencephalography), SPECT (single-photon emission computed tomography), fMRI (functional MRI).
