# Supplementary material for: Structural and Quantitative MRI Signal Features in Pediatric Focal Cortical Dysplasia: Clinical Relevance
Source: Physiol Res. 2026 Jun 1;75(3):597–612. doi: 10.33549/physiolres.935824 (PMC13557539; doi:10.33549/physiolres.935824)
Supplement: Supplementary file 4 [file 75_597_Suppl_Table_3.docx]

**Supplementary Table 3.** Summary of comparisons between contralateral and reference regions.

| *Tissue type* | **Sequences included** | **p-value range** | **Overall result** |
| --- | --- | --- | --- |
| *GM+WM* | T1w, T2w, FLAIR, qT1 | 0.48-0.88 | No significant differences across sequences |
| *WM* | T1w, T2w, FLAIR, qT1 | 0.41-0.85 | No significant differences across sequences |
| *GM* | T1w, T2w, FLAIR, qT1 | 0.48-0.98 | No significant differences across sequences |

GM – grey matter, WM – white matter.
